# Cross-Talk in Mechanomyographic Signals from the Forearm Muscles during Sub-Maximal to Maximal Isometric Grip Force

**DOI:** 10.1371/journal.pone.0096628

**Published:** 2014-05-06

**Authors:** Md. Anamul Islam, Kenneth Sundaraj, R. Badlishah Ahmad, Sebastian Sundaraj, Nizam Uddin Ahamed, Md. Asraf Ali

**Affiliations:** 1 AI-Rehab Research Group, Universiti Malaysia Perlis (UniMAP), Arau, Perlis, Malaysia; 2 Medical Officer, Malaysian Ministry of Health, Klang, Selangor, Malaysia; The University of Queensland, Australia

## Abstract

**Purpose:**

This study aimed: i) to examine the relationship between the magnitude of cross-talk in mechanomyographic (MMG) signals generated by the extensor digitorum (ED), extensor carpi ulnaris (ECU), and flexor carpi ulnaris (FCU) muscles with the sub-maximal to maximal isometric grip force, and with the anthropometric parameters of the forearm, and ii) to quantify the distribution of the cross-talk in the MMG signal to determine if it appears due to the signal component of intramuscular pressure waves produced by the muscle fibers geometrical changes or due to the limb tremor.

**Methods:**

Twenty, right-handed healthy men (mean ± SD: age  = 26.7±3.83 y; height  = 174.47±6.3 cm; mass  = 72.79±14.36 kg) performed isometric muscle actions in 20% increment from 20% to 100% of the maximum voluntary isometric contraction (MVIC). During each muscle action, MMG signals generated by each muscle were detected using three separate accelerometers. The peak cross-correlations were used to quantify the cross-talk between two muscles.

**Results:**

The magnitude of cross-talk in the MMG signals among the muscle groups ranged from, *R^2^_x, y_* = 2.45–62.28%. Linear regression analysis showed that the magnitude of cross-talk increased linearly (r^2^ = 0.857–0.90) with the levels of grip force for all the muscle groups. The amount of cross-talk showed weak positive and negative correlations (r^2^ = 0.016–0.216) with the circumference and length of the forearm respectively, between the muscles at 100% MVIC. The cross-talk values significantly differed among the MMG signals due to: limb tremor (MMG_TF_), slow firing motor unit fibers (MMG_SF_) and fast firing motor unit fibers (MMG_FF_) between the muscles at 100% MVIC (*p*<0.05, *η* ^2^ = 0.47–0.80).

**Significance:**

The results of this study may be used to improve our understanding of the mechanics of the forearm muscles during different levels of the grip force.

## Introduction

Surface mechanomyography (MMG) is a non-invasive technique, which records low frequency skin surface vibration caused by muscle contraction and is considered to be the mechanical equivalent to surface electromyography (sEMG) [1]. The MMG signals are influenced by changes in muscle force and length, and the relationship may be further complicated by changes in force levels from sub-maximal to maximal isometric contractions [2]. Although the relationship between the force and MMG signal has been used for the assessment of the conditions of muscle function [3], [4], some factors limit the applicability of the MMG technique for a comprehensive examination of muscle activity [5], [6]. For example, the cross-talk that occurs between adjacent muscles is one of the more important concerns associated with both MMG [5], [6] and sEMG techniques [7], [8].

In the field of EMG and MMG, cross-talk refers to the contamination of the signal from the muscle of interest by the signal from another muscle or muscle group that is in close proximity [9]. Consequently, many studies have investigated the cross-talk of sEMG signals (e.g., [7], [10], [11]). However, very few studies [6], [12] have analyzed cross-talk in MMG signals. Cramer *et al*. [12] quantified the cross-talk in MMG signals generated by the superficial quadriceps femoris muscles during maximal concentric and eccentric isokinetic muscle actions. In another study, Beck *et al*. [6] also examined cross-talk in MMG signals from the superficial quadriceps femoris muscles during sub-maximal to maximal muscle actions. These researchers [6] showed that the quadriceps femoris muscles generally provide independent activity and the exhibited cross-talk was inconsistent based on different levels of muscle action. However, these assessments may not be true for the forearm muscles because the forearm consists of many muscles in close proximity with varying degrees of common functions and because there is a relatively small area of the skin surface over these muscles for the placement of recording objects. Hence, it is expected that the forearm muscles will exhibit a higher degree of cross-talk than the leg muscles. In addition, the physiological interpretation of a signal generated by the forearm muscle of interest is difficult [7], [13].

To date, few studies have investigated the cross-talk in sEMG signals from the forearm muscles [7], [10], [11]. However, no study has carefully examined cross-talk with the MMG signal from the forearm muscles during different levels of muscle action. This is an important issue because the MMG signals are affected by muscle force, and this relationship is used in many applications [14], such as muscle function examination, prosthetic device control, and motor units control. Nonetheless, it is not clear whether the cross-talk in MMG signals changes in accordance with sub-maximal to maximal muscle actions of the grip force. Hypothetically, if cross-talk exists during muscle forces, then it should increase with the addition of muscle force because muscle activity increases with force. In addition, with an increment of force level the signal from each muscle becomes stronger.

However, the MMG signals due to the limb tremor need to be considered because the increasing force effort does not only transmit the tremor to nearby muscles but actually to the whole limb. For this reason a more precise evaluation of the MMG cross-talk need to be disclosed whether this cross-talk is the signal component due to intramuscular pressure waves produced by the motor unit fibers geometrical changes or due to the limb tremor. Under muscular contraction, mechanical vibrations occur due to three main processes [15]: (i) the inner muscular vibrations, which are the intrinsic components of the muscle contraction [16], (ii) oscillations of the human motor system, e.g. tremor and clonus [17], and (iii) artifacts. They are located in specific frequency ranges, with a certain amount of overlapping: most studies have used a filter with a 5 Hz high pass cutoff frequency to attenuate movement artifacts in MMG signals which is due to the influences of body and respiratory movements, as well as gross limb displacements [18]. The tremor frequency due to isometric contraction can go up to 12 Hz [19], [20], and the mechanical inner vibrations effect falls in the range between 10 and 40 Hz due to intrinsic muscle fibers oscillations [2], [16]. However, the entire frequency range of the MMG signal is widely defined between 5 and 100 Hz. It was hypothesized that the lower frequency band in the MMG signal refers to the firing rate of the slow twitching motor unit fibers whereas the higher frequency band of the signal displays the firing rate of the fast twitching motor unit fibers [21], [22]. Therefore, these signals are defined as MMG_TF_: the MMG signal due to tremor (5–12 Hz), MMG_SF_: the MMG signal due to slow firing motor unit fibers (12–40 Hz) and MMG_FF_: the MMG signal due to fast firing motor unit fibers (40–100 Hz) throughout the manuscript.

More importantly, the cross-talk in the MMG signals is not only dependent on just muscle effort but also on many other factors such as, the fiber composition, the distance between two muscles of interest, skin-fold thickness, and the level of activity of the two muscles [6], [23]. However, no previous study has investigated the cross-talk effect on the MMG signals as a function of muscle length and circumference.

Therefore, the purpose of this study were: i) to examine the relationship between the magnitude of cross-talk in the MMG signals generated by the forearm muscles with the sub-maximal to maximal isometric muscle actions of the grip force, and with the length and circumference of the forearm, and ii) to quantify the distribution of the cross-talk in the MMG signal to determine if it appears due to the signal component of intramuscular pressure waves produced by the muscle fibers geometrical changes or due to the limb tremor. However, one marked challenge of this experimentation is the measure and quantification of cross-talk. Although the application of cross-correlation functions has been criticized in previous studies [24], [25], it is currently the most powerful method for the quantification of cross-talk [6], [26]. The peak correlation coefficients (*R_x, y_*) at zero-phase shift are used as a cross-correlation function to quantify cross-talk. The cross-correlation coefficients which is the shared variance or percentage of common signal between two signals of adjacent muscle may be calculated by squaring the peak correlation to find the proportion of common signal (*R^2^_x, y_*  =  % cross-talk) between two muscles [10], [26].

## Materials and Methods

### Subjects

Twenty, healthy right-handed male volunteers (mean ± SD: age  = 26.7±3.83 y; height  = 174.47±6.3 cm; mass  = 72.79±14.36 kg; length of the forearm  = 26.95±1.47 cm; circumference of the forearm  = 26.65±2.44 cm) gave written consent prior to their participation in this experiment after being fully informed of the purpose of the investigation and the experimental protocols. All of the participants were clinically healthy with no previous or ongoing records of neuromuscular or skeletomuscular disorders specific to the elbow, wrist, and/or finger joints.

### Ethics

This study was approved (Ref No.: KKM/NIHSEP/P13-685) by the local Medical Research & Ethics Committee (MREC), Ministry of Health, Malaysia, and was performed in accordance with the principles of the Declaration of Helsinki.

### Muscle contraction protocols

**Figure 1:**
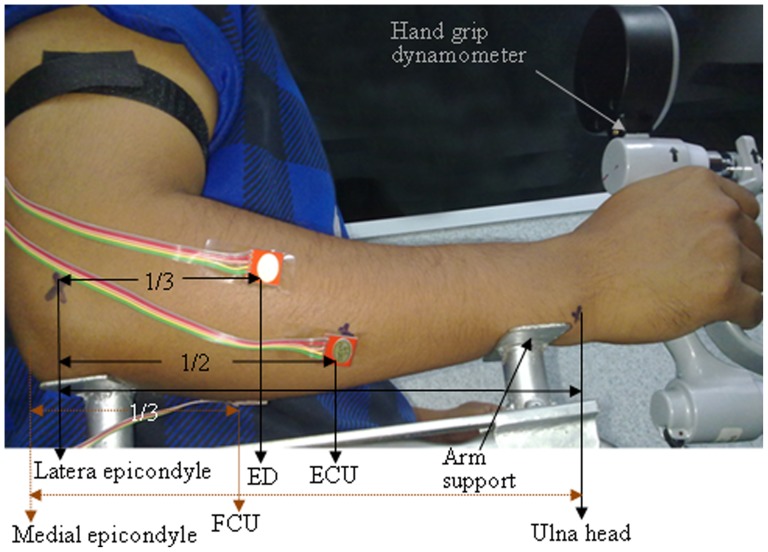
Schematic of an example for the placement of accelerometers used to detect the mechanomyography (MMG) signals from the bellies of extensor digitorum (ED), extensor carpi ulnaris (ECU) and flexor carpi ulnaris (FCU) muscles.

During the experiment, the subjects were seated comfortably on a chair with two adjustable arm supports attached to the chair arm. Each subject's forearm was placed on the arm supports with a neutral posture. The ulna bone positioned near the wrist and elbow (olecranon) joints was used to fix the arm supports at a height of 2 inches to ensure no contact pressure between the forearm muscles and the chair arm (Figure 1). Then the participants were requested to perform three trials of the maximum voluntary isometric contraction (MVIC) of the grip force. The participants were verbally encouraged to produce as much force as possible during each maximal trial. Each trial consisted of 6 s, and there was a resting period of 2 min between the trials. Of the three tests, the highest force was considered as the MVIC. After a resting period of 10 min, the participants were required to perform sub-maximal to maximal grip forces at approximately 20% increment of their maximum for 6 s each with a resting period of 2 min between the increments. The participants were shown visual feedback of the generated force and asked to maintain their force at the expected levels. The trials were repeated following the same resting period if any deviation of ±5% of their required forces was not achieved. All of the sub-maximal to maximal contractions was measured using a digital hand grip dynamometer (Digital Hand Dynamometer, SAEHAN Corporation, Korea). All of the muscle actions were performed at a joint angle of approximately 90° between the arm and the forearm. The distance between the medial epicondyle and distal head of ulna was considered as the length of the forearm. The circumference of the forearm was measured nearby the proximal part of the forearm, where the sensors were placed.

### MMG measurements

Three accelerometers (ADXL335, Analog Devices, USA; full-scale range  = ±3 g; typical frequency response  = 0.5–500 Hz; sensitivity  = 330 mV/g; size  = 15 mm×15 mm×1.5 mm, including breakout board on which it was mounted; weight including wires and board <1.5 gram) were used to detect the MMG signals. The three accelerometers were attached on the skin surface over the muscle bellies of the ED, ECU, and FCU with double-sided adhesive tape. The anatomical position of each muscle belly was determined according to the anatomical guide for the electromyographer by Perotto 2005 [27] as follows: ED – one third of the distance from proximal end of a line from lateral epicondyle of humerus to distal head of ulna; ECU – just lateral to ulnar border on the half way of the distance between the lateral epicondyle of humerus and distal head of ulna; FCU – two fingerbreadths from the ulnar border on one third of the distance between the medial epicondyle of humerus and distal head of ulna (Figure 1).

### Data acquisition and signal processing

The outputs of each of the three sensors were connected to the data acquisition unit (NI cDAQ 9191 wireless device and NI 9205 module with 16-bit resolution at CMMR of 100 dB, National Instruments, Austin, TX, USA), which differentially recorded the raw data at a rate of 1000 samples/s and stored the data in a computer for subsequent analyses. The raw data were bandpass-filtered (fourth-order Butterworth) at 5–100 Hz to obtain the MMG signals. Each MMG signal during the MVIC was then further passband-filtered (fourth-order Butterworth) at 5–12 Hz, 12–40 Hz and 40–100 Hz to obtain the MMG_TF_, MMG_SF_ and MMG_FF_ signals, respectively. The MMG signals were extracted for a 2-s period corresponding to the middle 33% of each 6-s muscle action. The 2-s segments for each MMG signal were used to perform the cross-correlation. The cross-correlation values of the two signals *X_t_* and *Y_t_* were determined according to the following equation:
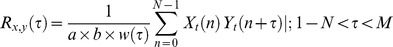
(1)where 
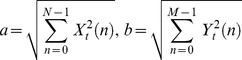
, *w* is the weighting factor, *M* and *N* are the lengths of *X_t_* and *Y_t_*, respectively, and τ represents the time lag between the signals. The peak cross-correlation coefficients were squared to obtain the magnitude of the cross-talk, *R* ^2^ *_x, y_* (common signal %) between the two MMG signals generated by different muscles in the muscle group. All of the signal processing was performed with custom programs written in the LabVIEW programming software (version 12.0, National Instruments, Austin, TX, USA).

### Data analysis

Linear regression was used to observe the relationship between different levels of the grip force and the cross-talk and between the anthropometric parameters of the forearm and the magnitude of cross-talk in the MMG signals. One-way Analysis of Variance (ANOVA) tests were used among the cross-talk values in the MMG signals between the muscle groups that were investigated. The statistical analyses were performed using Minitab (Minitab 14, Minitab Inc, PA, USA) and Microsoft Excel 2007 software tools. The critical value of F-ratio, *F*_c_ = 3.15 at a significance level of α = 0.05 was affixed for statistical significant analysis. Therefore, any value of *p*≤0.05 was considered statistically significant.

## Results

**Figure 2:**
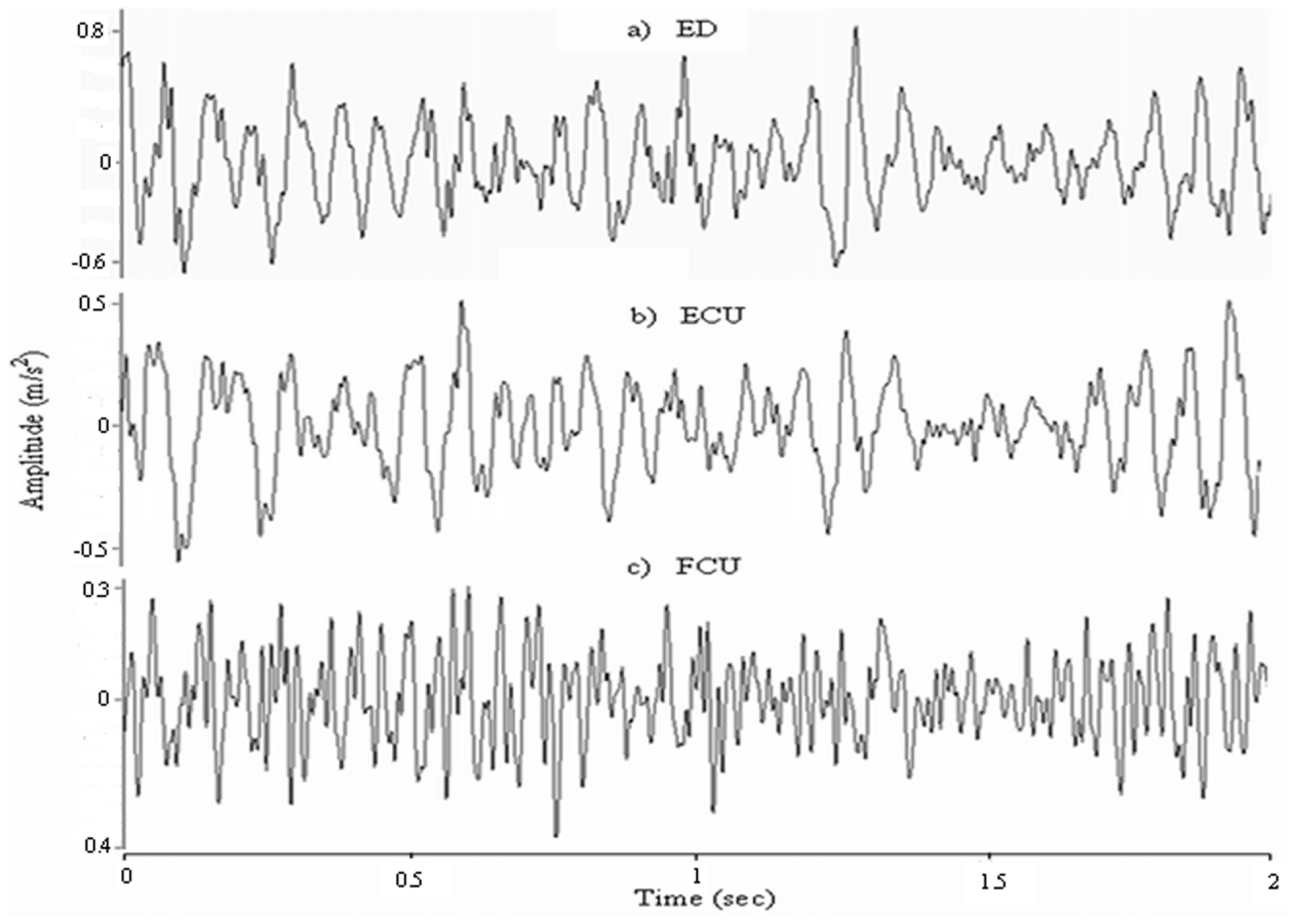
The mechanomyographic (MMG) signals from: a) extensor digitorum (ED), b) extensor carpi ulnaris (ECU), and c) flexor carpi ulnaris (FCU) muscles at 100% MVIC, which were used to analyze cross-talk.

**Figure 3:**
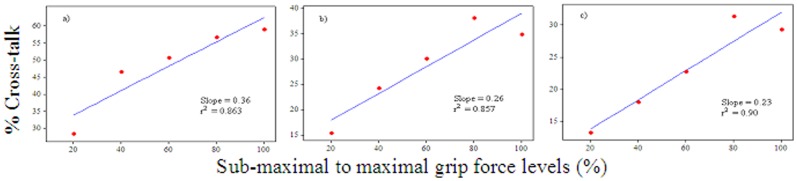
Correlation between the sub-maximal to maximal isometric contractions of the grip force and amount of the cross-talk (i.e., % common signal between two muscles) in the MMG signals between: a) ED and ECU, b) ECU and FCU, and c) FCU and ED muscle groups.

**Figure 4:**
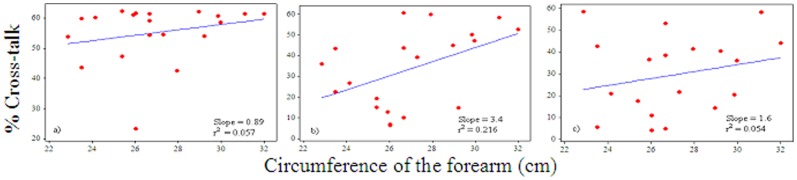
Correlation between circumference of the forearm and amount of the cross-talk in the MMG signals between: a) ED and ECU, b) ECU and FCU, and c) FCU and ED muscle groups at 100% MVIC.

**Figure 5:**
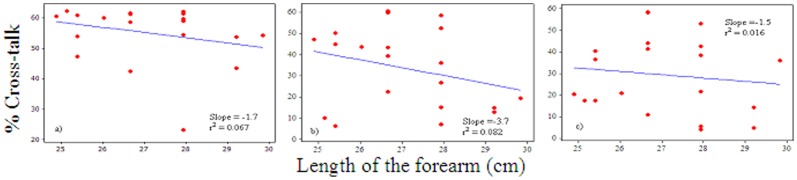
Correlation between length of the forearm and amount of the cross-talk in the MMG signals between: a) ED and ECU, b) ECU and FCU, and c) FCU and ED muscle groups at 100% MVIC.

Figure 2 shows an example of the extracted MMG signals generated by the ED, ECU and FCU muscle groups at 100% MVIC for one subject. Tables 1–3 show the magnitude of cross-talk in the MMG signals between the ED and ECU, ECU and FCU, as well as FCU and ED muscle groups, which were obtained for each subject and each force level. The magnitude of the overall cross-talk in the MMG signals for all of the conditions ranged from *R* ^2^ *_x, y_* = 2.45–62.28%. There were strong positive correlations between the levels of the grip force and the magnitude of cross-talk in the MMG signals between the ED and ECU (r^2^ = 0.863, m = 0.36), ECU and FCU (r^2^ = 0.857, m = 0.26) and FCU and ED (r^2^ = 0.90, m = 0.23) muscle groups; m representing slope in linear regression lines (Figure 3). There were weak positive correlations between the circumference of the forearm and the amount of cross-talk in the signals between the ED and ECU (r^2^ = 0.057; m = 0.89), ECU and FCU (r^2^ = 0.216%; m = 3.4) as well as FCU and ED (r^2^ = 0.054; m = 1.6) muscle groups during 100% MVIC (Figure 4). However, there were weak negative correlations between the length of the forearm and the amount of cross-talk in the signals between the ED and ECU (r^2^ = 0.067; m = −1.7), ECU and FCU (r^2^ = 0.082%; m = −3.7) as well as FCU and ED (r^2^ = 0.016; m = −1.5) muscle groups during 100% MVIC (Figure 5).

**Table 1:** The magnitude of cross-talk in MMG signals between the ED and ECU muscle pairs for different levels of the grip force.

Subjects	% Cross-talk at different MVIC levels				
	20%	40%	60%	80%	100%
1	12.62	27.86	52.04	60.35	60.66
2	43.59	55.04	53.34	59.79	62.28
3	21.56	61.98	50.07	36.34	54.06
4	34.63	42.11	52.24	51.41	61.05
5	10.62	18.70	50.60	60.96	47.39
6	43.60	50.95	52.74	60.80	60.16
7	38.10	42.33	56.88	56.12	61.59
8	50.25	41.57	52.69	60.90	58.69
9	41.61	50.79	55.94	59.38	61.51
10	13.38	42.19	53.74	59.31	61.36
11	30.58	39.19	45.95	32.04	42.47
12	31.07	59.40	53.89	60.72	61.51
13	24.23	48.72	51.68	61.97	59.82
14	7.06	18.94	29.04	28.97	54.52
15	48.94	58.74	55.66	60.34	62.14
16	11.53	26.42	57.75	61.10	59.08
17	6.53	9.50	25.67	15.13	23.23
18	36.55	35.92	40.29	46.29	53.90
19	16.83	42.17	30.63	62.04	43.65
20	25.82	31.21	35.03	60.86	54.29

**Table 2:** The magnitude of cross-talk in MMG signals between the ECU and FCU muscle pairs for different levels of the grip force.

Subjects	% Cross-talk at different MVIC levels				
	20%	40%	60%	80%	100%
1	7.57	17.55	50.89	56.39	50.26
2	5.35	3.70	10.28	14.50	19.38
3	5.01	11.81	7.77	15.71	14.81
4	15.39	6.48	23.83	18.11	12.88
5	4.25	12.09	5.20	28.03	15.07
6	9.01	26.39	31.67	23.53	26.70
7	30.24	20.40	26.04	17.37	6.96
8	15.11	8.00	43.93	29.60	35.96
9	29.96	47.61	56.33	57.84	58.45
10	26.97	34.51	24.04	49.06	52.58
11	31.97	54.50	53.83	60.99	59.90
12	36.36	55.97	53.13	60.17	60.62
13	12.25	30.16	59.29	60.89	43.35
14	2.89	11.27	13.53	18.22	39.35
15	17.76	38.84	33.64	48.94	22.46
16	12.98	18.38	27.64	52.61	43.78
17	9.62	15.17	6.40	14.37	6.30
18	15.11	39.37	17.78	27.45	44.87
19	4.11	6.69	7.91	7.44	10.16
20	14.83	24.91	28.11	49.45	47.24

**Table 3:** The magnitude of cross-talk in MMG signals between the ED and FCU muscle pairs for different levels of the grip force.

Subjects	% Cross-talk at different MVIC levels				
	20%	40%	60%	80%	100%
1	8.73	7.19	45.53	52.52	20.33
2	6.63	5.71	10.95	11.10	17.56
3	3.32	8.78	19.59	35.08	40.40
4	15.36	4.00	23.24	40.05	36.40
5	2.45	5.48	8.72	35.69	17.58
6	8.34	20.66	28.69	24.22	20.90
7	11.01	17.33	13.01	16.86	10.91
8	13.75	7.37	24.59	56.23	58.40
9	12.87	30.68	40.72	54.23	58.31
10	27.29	20.21	11.35	35.98	44.18
11	43.58	51.98	37.33	36.77	41.31
12	19.31	38.14	49.26	45.25	53.18
13	5.70	21.08	40.64	59.00	42.62
14	3.84	5.19	6.32	6.92	21.59
15	33.83	36.46	15.58	19.77	5.45
16	10.50	12.52	14.38	26.37	38.49
17	6.79	4.22	5.19	3.56	4.09
18	14.55	19.08	11.21	13.29	14.19
19	3.92	5.90	17.18	3.09	4.78
20	12.37	37.16	31.96	21.67	35.97

Tables 4–6 show the magnitude of the cross-talk in the MMG_TF_, MMG_SF_ and MMG_FF_ signals generated by the muscle groups at 100% MVIC. The MMG_TF_ (range: 11.09–95.17%) and MMG_FF_ (range: 2.18–26.10%) signals showed higher and lower cross-talk values, respectively for all the muscle groups (Tables 4 and 6). The cross-talk (range: 6.12–66.24%) also occurred in the MMG_SF_ signals for all the muscle groups (Table 7). We also found that there were statistically significant differences in the cross-talk values among all the MMG signals due to tremor, slow and fast firing motor unit fibers for any of the muscle groups at 100% MVIC (Table 7) (*F* = 113.44, *p* = 0.0001, *η^2^* = 0.80 between the ED and ECU; *F* = 30.74, *p* = 0.0001, *η^2^* = 0.52 between the ECU and FCU; *F* = 25.59, *p* = 0.0001, *η^2^* = 0.47 between ED and FCU muscle groups).

**Table 4:** The magnitude of cross-talk in the MMG_TF_ signals between the muscle groups at 100% MVIC.

Subjects	% Cross-talk		
	ED-ECU	ECU-FCU	FCU-ED
1	80.27	56.48	21.09
2	87.59	37.33	44.68
3	84.19	62.08	33.15
4	84.60	11.09	23.08
5	80.71	46.94	48.25
6	90.59	34.69	21.93
7	76.68	25.84	18.19
8	94.30	88.63	81.58
9	22.93	86.92	26.60
10	53.13	14.70	19.26
11	85.78	92.77	69.78
12	95.17	88.74	81.69
13	67.08	90.99	56.78
14	89.19	84.33	78.30
15	86.13	59.61	68.26
16	67.40	58.68	21.04
17	76.25	62.76	29.96
18	73.31	18.44	14.25
19	91.34	80.82	76.71
20	78.09	27.81	26.22

**Table 5:** The magnitude of cross-talk in the MMG_SF_ signals between the muscle groups at 100% MVIC.

Subjects	% Cross-talk		
	ED-ECU	ECU-FCU	FCU-ED
1	64.56	51.26	24.54
2	64.28	6.16	6.44
3	37.50	6.30	23.63
4	66.24	20.16	41.27
5	55.70	19.07	23.03
6	57.24	24.38	9.92
7	50.27	27.16	25.75
8	53.95	54.08	21.41
9	31.09	65.50	19.96
10	16.56	8.62	32.20
11	58.28	61.87	50.23
12	63.61	49.89	42.88
13	37.25	66.13	40.72
14	65.89	63.39	53.10
15	57.17	22.98	22.67
16	31.17	52.50	14.90
17	64.57	50.34	36.63
18	14.65	8.94	6.12
19	49.19	40.19	31.00
20	35.54	18.55	9.83

**Table 6:** The magnitude of cross-talk in the MMG_FF_ signals between the muscle groups at 100% MVIC.

Subjects	% Cross-talk		
	ED-ECU	ECU-FCU	FCU-ED
1	16.17	8.24	10.70
2	5.30	11.96	3.05
3	3.11	4.71	2.18
4	2.97	12.19	6.61
5	10.47	3.95	3.81
6	13.17	6.47	4.30
7	26.10	5.11	5.15
8	16.24	3.75	4.85
9	17.72	8.51	5.08
10	4.86	3.72	3.59
11	3.87	3.46	2.98
12	3.69	4.47	4.67
13	6.19	3.50	3.78
14	7.57	7.36	6.12
15	12.21	3.09	5.29
16	19.81	6.71	19.88
17	4.00	3.76	3.41
18	5.73	5.73	6.23
19	25.55	6.87	6.09
20	14.85	5.12	7.15

**Table 7:** Statistical analysis of the mean cross-talk among the MMG_TF_, MMG_SF_ and MMG_FF_ signals between the muscle groups at 100% MVIC.

Muscle pairs	MMG bands	Mean (SD)	Mean square	Standard error	*F*	*p*-value	Effect size, *η^2^*
ED & ECU	MMG_TF_	78.24 (16.61)	22732.70	4.48	113.44	0.0001	0.80
	MMG_SF_	48.73 (16.43)					
	MMG_FF_	10.98 (7.44)					
ECU & FCU	MMG_TF_	56.48 (28.01)	12921.59	6.48	30.74	0.0001	0.52
	MMG_SF_	35.87 (21.67)					
	MMG_FF_	5.93 (2.66)					
ED & FCU	MMG_TF_	43.04 (24.68)	6991.60	5.23	25.59	0.0001	0.47
	MMG_SF_	26.81 (14.00)					
	MMG_FF_	5.75 (3.83)					

## Discussion

The present study quantified and correlated the magnitude of the cross-talk in MMG signals between the ED, ECU, and FCU muscles during the sub-maximal to maximal isometric muscle actions of the grip force. This study also examined the relationship between the amount of cross-talk in the MMG signals and the anthropometric parameters of the forearm during 100% MVIC. Furthermore, this study analyzed the cross-talk in the MMG signals due to the tremor, slow and fast firing motor unit fibers. In all of the measured cross-correlations, almost all of the peak coefficients appeared at a time shift of approximately 0 s (i.e., τ = 0 s). Cross-talk was observed in the MMG signals generated by the forearm muscles for all of the conditions that were performed. The magnitude of cross-talk in the MMG signals ranged from, *R* ^2^ *_x, y_* = 2.45% to 62.28% considering all of the conditions that were performed (Tables 1–3). In addition, the cross-talk also appeared in all the MMG_TF_ (range: 11.09–95.17%), MMG_SF_ (range: 6.12–66.24%) and MMG_FF_ (range: 2.18–26.10%) signals between the muscle groups during 100% MVIC. This assessment supports the observation that the complete differentiation of muscle activities of the different forearm muscles is difficult [13]. This may be because there are more than ten individual muscles in the forearm, which act to flex and extend the phalanges and hand (e.g., extensor carpi radialis longus, extensor carpi radialis brevis, extensor carpi ulnaris, extensor pollicis brevis, flexor carpi ulnaris, and flexor pollicis longus). Together, these muscles may contribute to the MMG signals due to their close proximity and the small surface area to which the sensors were placed.

Despite the intricacy of association among studies because of diverse experimental conditions and cross-talk quantification indices, the magnitude of the cross-talk obtained in this study can be compared to the findings reported by Beck *et al*. [6] who found that the peak correlation coefficients between the superficial quadriceps femoris muscles ranged from 0.124 to 0.714 (i.e., 1.5 to 51% common signal) during sub-maximal to maximal isometric contractions. As expected, the forearm muscles experienced higher cross-talk compared with the quadriceps muscle groups because the forearm muscles are relatively smaller in size and thus closer in proximity which may be influenced to generate the greater cross-talk. Kong et al. [7] revealed that the amounts of cross-talk with sEMG generally ranged from 4 to 50% in the forearm flexors. In another study, Mogk and Keir [11] reported that the amount of cross-talk in sEMG signals were up to 64% for flexors and 58% for extensors during gripping tasks. The findings of these studies [7], [11] may also be compared to the findings of the present study.

The present study also observed that there were strong positive correlations between the different levels of the grip force and the amount of cross-talk in the MMG signals between the between the ED and ECU, ECU and FCU, as well as FCU and ED muscle groups. The slopes for the cross-talk between the ED and ECU (m = 0.36), ECU and FCU (m = 0.26) as well as FCU and ED (m = 0.23) muscle groups indicate that an increment of 20% grip force also increased the amount of cross-talk by 7.2%, 5.2% and 4.6%, respectively. This assessment supports the findings reported by Solomonow *et al*. [28] who reported that the cross-talk with the wire EMG between the lateral gastrocnemius and tibialis anterior increased linearly during increasing force contraction from 10 to 100% of the maximal force. However, the findings of the present study is contrary to the findings reported by Beck *et al*. [6] who examined the quadriceps femoris muscles and described that “………., and the cross-correlation coefficients generally did not increase with isometric torque”.

It can be pointed out that an increment of 20% force level refers to orderly recruitment of the motor unit in the MMG signals. Therefore, the results of this study have practical meaning, specifically in the application of MMG technique for the examination of motor control and muscle mechanics during various degrees of muscle action. Hence, the amount of cross-talk needs to be accounted when different levels of the force measurement are of interest. Further, there were weak positive correlations between the circumference of the forearm and the amount of cross-talk in the MMG signals between the ED and ECU, ECU and FCU as well as FCU and ED muscle groups at 100% MVIC. This assessment is contrary to the finding reported by Yung and Wells [10], who stated that “Individuals with larger forearm would be expected to push the cross-correlations towards lower values while higher correlations would be seen in the smaller individuals”. The results that the present study observed are possibly due to the fact that most of the forearms considered in this experiment were large due to larger musculature, which might produce larger cross-talk values instead of larger forearms due to higher levels of fat, which may attenuate the MMG signal and consequently produce smaller cross-talk values. However, there were weak negative correlations between the length of the forearm and the amount of cross-talk in the signals between the ED and ECU, ECU and FCU as well as FCU and ED muscle groups during 100% MVIC. The slopes indicate that 1 cm changes in the forearm length decreased the cross-talk values by 1.7%, 3.7% and 1.5% from the MMG signals between the ED and ECU, ECU and FCU and FCU and ED muscle groups respectively (Figure 5). This is possibly due to the fact that the muscle contraction originates from the proximal part and attenuates throughout the propagation path towards the distal end and is thus expected to lower the cross-talk values in longer forearms. It is interesting to point out that there are other factors such as muscle fibers composition, skin-fold thickness, and distance between the muscles of interest that may equally affect the MMG signal other than the size and length of the forearm [6], [23].

We also found that there were statistically significant differences in the cross-talk values among the MMG_TF_, MMG_SF_ and MMG_FF_ signals in the muscle groups at 100% MVIC. According to Cohen's interpretation of effect size for *F*-ratio statistics when *p*≤0.05, Eta Squared (*η^2^*) is used to determine effect size: 0.01 =  small, 0.06 =  medium and 0.138 =  large [29]. Thus, the present study observed a large effect size on the cross-talk values among the different bands of the MMG signals between the muscle groups at 100% MVIC (Table 7).

*Post-hoc* analysis was used to analyze the statistical significance of the cross-talk values between each individual groups of the three different band MMG signals (i.e., between the MMG_TF_ and MMG_SF_; between the MMG_SF_ and MMG_FF_ and between the MMG_TF_ and MMG_FF_). For all the cases the mean differences of the cross-talk values were much higher than the related standard error. Therefore, this study further confirmed that the amount of cross-talk significantly differed among the MMG_TF_, MMG_SF_ and MMG_FF_ signals at 100% MVIC for all the muscle groups that were investigated.

The present study has several possible limitations. This study did not consider the skin-fold thickness of the forearm muscles, which cannot be ruled out because tissue thickness (e.g., subcutaneous fat, skin, and bone) influences the amount of cross-talk in both MMG and sEMG signals [23], [28]. This study also did not consider muscle fatigue and/or stiffness due to repeatedly produced muscle force. It is expected that the rest breaks between trials performed in this study may reduce these effects. In addition, the present study also used cross-correlation based index rather than amplitude based because the former is easier to use in practice, since it can quantify the amount of cross-talk between two muscles without collecting information of the contaminated signal. However, cross-correlation does not necessarily always mean cross-talk. There is evidence that the cross-correlation method being used to determine motor unit synchrony as common neural input between two muscles [30]. If two muscles receive common input and response similarly with the muscle action, then it is reasonable to expect that they would exhibit high cross-correlations instead of cross-talk. Therefore, cross-correlations can represent cross-talk, but it can also represent other mechanisms. It is also documented that both amplitude- and correlation-based indices were not statistically significant in the case of cross-talk magnitude quantification [25]. Therefore, the results of this study may be used in certain applications where precise measurements of motor unit control are essential, such as externally powered prosthetics.

## Conclusions

In summary, the present study observed the following: First, the MMG signals generated by the forearm muscles exhibited cross-talk regardless of the levels of the grip force that were performed. The cross-talk also appeared in the MMG signals oscillated only by the muscle motor unit fibers regardless of the tremor signal. Thus, it can be concluded that MMG signals cannot be used to completely differentiate the activities of the forearm muscles during sub-maximal to maximal muscle contractions. Second, the magnitude of cross-talk in the MMG signals showed strong positive correlation with the different levels of the grip force for all the muscle groups that were examined. Therefore, the amount of cross-talk in the MMG signals needs to be accounted when the condition of muscle function measurements using different levels of the grip force from the forearm muscles are concerned. Third, the amount of cross-talk was also influenced by the circumference and length of the forearm for all the muscle groups. Therefore, anthropometric parameters of the forearm may influence the amount of cross-talk in the MMG signals. Further investigation is needed to determine the cross-talk effects on the MMG signals from different muscle groups using various types of static and dynamic muscle actions.
